# Room Temperature Ionic Liquid Capping Layer for High Efficiency FAPbI_3_ Perovskite Solar Cells with Long‐Term Stability

**DOI:** 10.1002/advs.202400117

**Published:** 2024-03-13

**Authors:** Qiang Lou, Xinxin Xu, Xueqing Lv, Zhengjie Xu, Tian Sun, Liwen Qiu, Tingting Dai, Erjun Zhou, Guijun Li, Tong Chen, Yen‐Hung Lin, Hang Zhou

**Affiliations:** ^1^ School of Electronic and Computer Engineering Peking University Shenzhen Graduate School Shenzhen 518055 China; ^2^ CAS Key Laboratory of Nanosystem and Hierarchical Fabrication CAS Center for Excellence in Nanoscience National Center for Nanoscience and Technology Beijing 100190 China; ^3^ Key Laboratory of Optoelectronic Devices and Systems of Ministry of Education and Guangdong Province College of Physics and Optoelectronic Engineering Shenzhen University Shenzhen 518060 China; ^4^ Department of Electronic and Computer Engineering The Hong Kong University of Science and Technology Hong Kong SAR 999077 P. R. China

**Keywords:** ionic liquid, passivation, perovskite, solar cells, stability

## Abstract

Ionic liquid salts (ILs) are generally recognized as additives in perovskite precursor solutions to enhance the efficiency and stability of solar cells. However, the success of ILs incorporation as additives is highly dependent on the precursor formulation and perovskite crystallization process, posing challenges for industrial‐scale implementation. In this study, a room‐temperature spin‐coated IL, n‐butylamine acetate (BAAc), is identified as an ideal passivation agent for formamidinium lead iodide (FAPbI_3_) films. Compared with other passivation methods, the room‐temperature BAAc capping layer (BAAc RT) demonstrates more uniform and thorough passivation of surface defects in the FAPbI_3_ perovskite. Additionally, it provides better energy level alignment for hole extraction. As a result, the champion n–i–p perovskite solar cell with a BAAc capping layer exhibits a power conversion efficiency (PCE) of 24.76%, with an open‐circuit voltage (Voc) of 1.19 V, and a Voc loss of ≈330 mV. The PCE of the perovskite mini‐module with BAAc RT reaches 20.47%, showcasing the effectiveness and viability of this method for manufacturing large‐area perovskite solar cells. Moreover, the BAAc passivation layer also improves the long‐term stability of unencapsulated FAPbI_3_ perovskite solar cells, enabling a T80 lifetime of  3500 h when stored at 35% relative humidity at room temperature in an air atmosphere.

## Graphical abstract

In this study, a room‐temperature spin‐coated IL, n‐butylamine acetate (BAAc), is identified as an ideal passivation agent for formamidinium lead iodide (FAPbI_3_) films. The PCE of the devices treated by BAAc RT achieves 24.76%, with a Voc value reaching as high as 1.19 V.

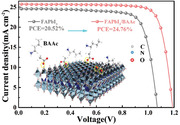

## 1 Introduction

Since the invention of perovskite solar cells (PSCs) in 2009,^[^ ^1^ ^]^ their power conversion efficiency (*PCE*) has progressively increased from 3.8% to 26.1%.^[^ ^2^, ^3^, ^4^ ^]^ The recent advancements in PSCs are attributed to the passivation of defects in perovskite thin films and the suppression of their non‐radiative recombination. Defects in perovskite films, including halide vacancies, incongruous Pb^2+^, and Pb–I vacancies, form trap states leading to non‐radiative recombination.^[^ ^5^, ^6^, ^7^ ^]^ The non‐radiative recombination often results in suboptimal interfacial energy configurations and defect states,^[^ ^8^ ^]^ affecting the open‐circuit voltage (*Voc*) performance of the device. Thus, efficient PSCs necessitate the effective suppression of non‐radiative recombination.^[^ ^9^, ^10^ ^]^

Interface engineering has proven effective in reducing defects in perovskite films, thereby improving the performance and stability of PSCs.^[^ ^11^ ^]^ The construction of 2D/3D heterojunction perovskites is considered an effective approach to prevent degradation pathways.^[^ ^12^, ^13^ ^]^ Numerous alkyl ammonium spacer cations, including butyl ammonium (C_4_H_12_N^+^; BA^+^),^[^ ^14^ ^]^ octylamine (C_8_H_20_N^+^; OA^+^),^[^ ^15^ ^]^ phenylammonium (C_6_H_5_NH_3_^+^; PEA^+^),^[^ ^16^ ^]^ and oleoamine (C_18_H_38_N^+^; OLA ^+^),^[^ ^17^ ^]^ have been widely reported for interfacial modification. However, due to the quantum‐limiting effect of 2D perovskites, the 2D/3D double‐layer structure often suffers from low charge transport efficiencies.^[^ ^18^ ^]^ Additionally, the uneven distribution of the 2D perovskite layer significantly limits the reproducibility of devices over large areas.^[^ ^19^, ^20^ ^]^ Therefore, there is a strong desire to directly treat the perovskite surface with aryl ammonium salts, rather than the corresponding 2D perovskite, to enhance device performance. In 2019,^[^ ^16^ ^]^ You et al. passivated conventional perovskite (≈ 1.53 eV) surfaces directly with phenylammonium iodide (PEAI) instead of PEA_2_PbI_4_, resulting in an ultra‐high certified *PCE* of 23% and a *Voc* of 1.18 V. This impressive result has sparked interest in applying this direct passivation strategy to other types of perovskite devices for enhanced performance. Recently, Yang et al. found that when the surface of perovskite was passivated with ammonium iodide (octylium ammonium iodide, OAI), fixing OA^+^ as the cation and replacing I^−^ with other anions (benzenesulfonic acid, TsO^−^), not only could the energy level arrangement of the device be effectively adjusted, but also the migration of ions at the interface could be reduced.^[^ ^15^ ^]^ Nonetheless, the exploration of anions of capping materials still lags behind their cation counterparts.

Ionic liquid salts (ILs) represent molten salts that maintain a liquid state at room temperature, showcasing notable characteristics such as high ionic conductivity, hydrophobicity, thermodynamic and thermal stability, and chemical durability.^[^ ^21^, ^22^, ^23^ ^]^ Many studies have previously applied ILs in solar cells,^[^ ^24^, ^25^, ^26^, ^27^ ^]^ primarily as additives in perovskite precursor solutions, aiming to improve material quality and stability. However, the use of ILs as additives in perovskite solutions is highly sensitive to perovskite formulations and coating technologies, posing challenges for industrialization.^[^ ^23^, ^27^ ^]^ Recently, researchers have introduced ILs based on 1‐ethyl‐3‐methylimidazole (EMIM^+^) cations as passivation layers for n–i–p and p–i–n PSCs. Zhu et al. anchored Pb^2+^ and I^−^ to the surface of FAPbI_3_ by an in situ reaction with selected 1‐ethyl‐3‐methylimidazolium bromide ([EMIM]Br) IL, forming a thin ionic‐liquid‐perovskite capping layer. ILs can stabilize the perovskite by establishing strong chemical bonds with the soft perovskite film, effectively preventing the loss of perovskite components and suppressing the density at grain boundaries and interfaces of capture states. As a result, this passivation effect improves both the efficiency and operational stability of the PSCs.^[^ ^28^ ^]^ Huang et al. utilized EMIM^+^ cations of ILs along with different halide anions (Cl^−^, Br^−^, and I^−^) in inverted PSCs. Employing an in‐situ growth technology, EMIMX forms a 1D passivation layer influencing the surface morphology of the perovskite film. These EMIMX‐treated layers simultaneously inhibit surface defects and non‐radiative energy loss while improving hydrophobicity.^[^ ^29^ ^]^

In this study, an ionic liquid salt, n‐butylamine acetate (BAAc), is introduced as a multifunctional interlayer material coated onto pre‐crystallized perovskite layers to concomitantly improve the efficiency and stability of PSCs. Employing the multifunctional modification strategy, a room‐temperature‐treated BAAc ultra‐thin layer (denoted as BAAc RT) is deposited on the top surface of the FAPbI_3_ perovskite layer. Comparing with the conventional multidimensional (2D/3D) heterojunction perovskite formed by thermally annealing BAAc (denoted as BAAc TA), the room‐temperature coating technique significantly lowers the number of uncoordination defects on the FAPbI_3_ film surface and optimizes the energy level arrangement, thereby inhibiting non‐radiative complexation pathways. Furthermore, the BAAc RT and the BAI RT (treatment involving n‐butylammonium iodide at room temperature) capping layers were systematically compared. The findings reveal that BAAc RT improves the homogeneous surface morphology of the perovskite film and outperforms its iodized salt counterparts in terms of surface passivation. The device treated with BAAc RT achieved an outstanding *PCE* of 24.76% with a high *Voc* value of 1.19 V. This performance considerably exceeds that of the untreated device (20.53% for *PCE* and 1.08 V for *Voc*). The application of this strategy in manufacturing mini‐modules (5 × 5 cm^2^, active area of 12 cm^2^) showed a substantial increase in their active area efficiency from 17.39% to 20.47%.

## 2 Results and Discussion

**Figure 1:**
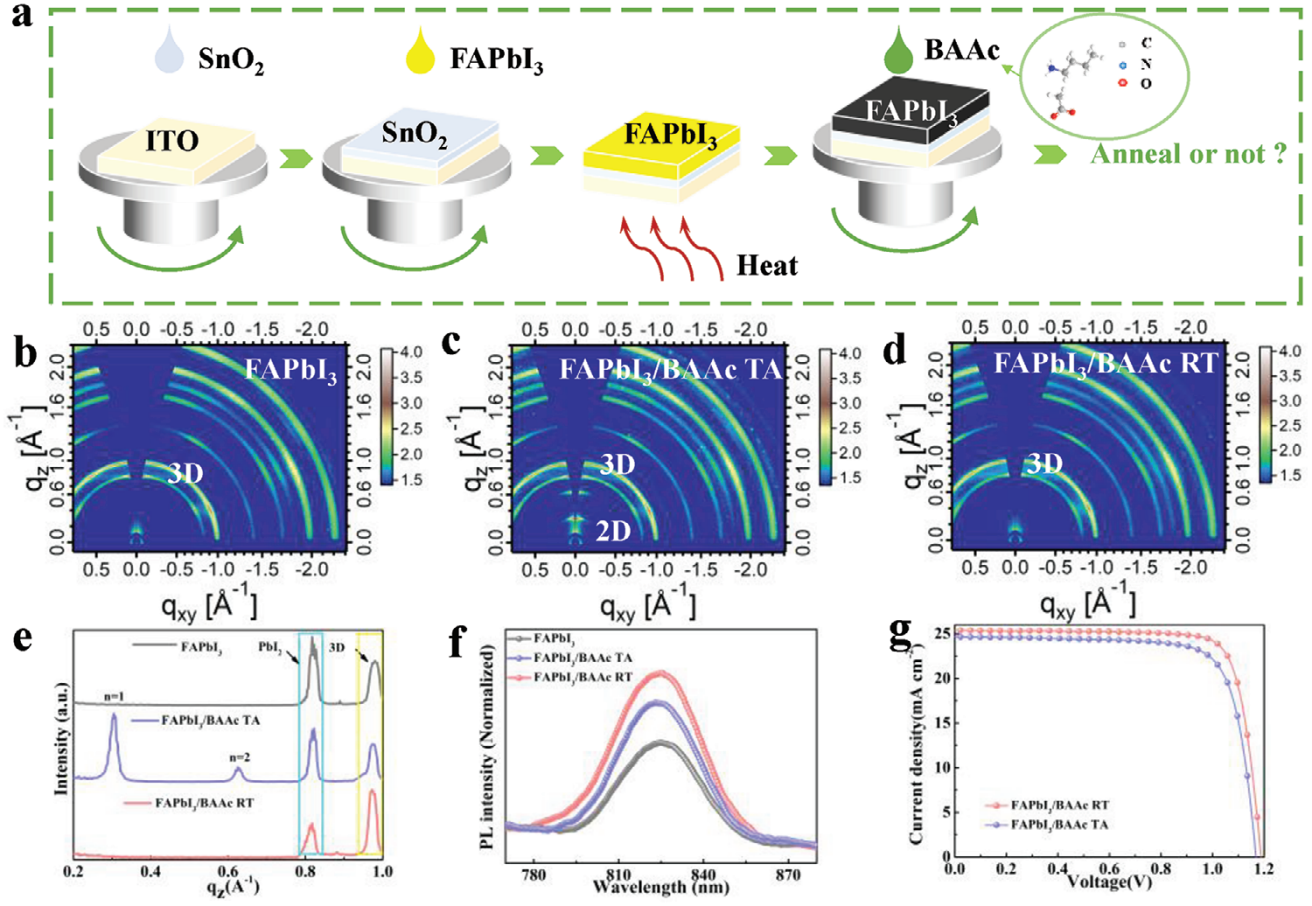
a) The technical route for fabricating BAAc‐modified FAPbI_3_ film. Grazing‐incidence wide‐angle X‐ray scattering (GIWAXS) images for b) FAPbI_3_ c) FAPbI_3_/BAAc TA (Temperature Annealing, 100 °C) and d) FAPbI_3_/BAAc RT (Room Temperature, 25 °C) films. e) Cut‐lines of GIWAXS intensities along the q_z_ axis. f) PL spectral curve. g) *J*–*V* curves of the champion devices based on different perovskite films.

**Figure** **1**a shows the one‐step spin‐coating method used to prepare perovskite films, with the chemical structure of BAAc shown in the accompanying illustration. In the post‐treatment at the interface, isopropyl alcohol (IPA) solutions with varying ratios (volume ratios) of BAAc contents were coated on the surface of the pre‐crystallized perovskite film. Before the fabrication of PSCs, it is necessary to investigate the effect of the post‐annealing step on the BAAc–perovskite interface. In the perovskite film preparation process, the FAPbI_3_ halide perovskite precursor solution was deposited in a mixed solvent of N, N‐dimethylformamide (DMF), and dimethylsulfoxide (DMSO) by a one‐step spin‐coating method (refer to the Experimental Section). Three types of perovskite thin films were fabricated for comparison: perovskite without BAAc, perovskite with BAAc at room temperature (denoted as BAAc RT), and perovskite with BAAc post‐annealed at 100 °C (denoted as BAAc TA).

The scattering pattern in Figure 1b shows that the crystal orientation of 2D perovskites after BAAc TA treatment is vertical, in contrast to the random crystal orientation observed in 3D polycrystalline perovskites (typical ring at 0.97 A^−1^). The cut line along the q_z_ axis displays two additional scattering vectors at 0.43 and 0.62 A^−1^, indexed as (020) (*n* = 1) and (040) (*n* = 2), respectively.^[^ ^30^, ^31^ ^]^ Notably, FAPbI_3_/BAAc does not exhibit the crystal orientation characteristic of 2D perovskites at room temperature, indicating that the crystal orientation of perovskite films is significantly influenced by the annealing process. The presence of a 2D perovskite layer after annealing treatment of FAPbI_3_/BAAc RT was confirmed by X‐ray techniques, and its orientation was determined using grazing‐incidence wide‐angle scattering (GIWAXS). Upon comparing the peak strength of PbI_2_ in FAPbI_3_, FAPbI_3_/BAAc RT, and FAPbI_3_/BAAc TA films, it was observed that annealing resulted in the formation of a substantial amount of PbI_2_, while FAPbI_3_/BAAc RT films exhibited the least PbI_2_ (Figure S1, Supporting Information).^[^ ^32^ ^]^ Therefore, the BAAc RT treatment yields the highest crystallinity quality among the three conditions.

The steady‐state photoluminescence (PL) and time‐resolved photoluminescence (TRPL) spectra of perovskite films deposited on glass substrates before and after surface modification were analyzed to reveal the correlations between film defects and carrier dynamics before and after annealing. As shown in Figure 1f, compared to the control device, the luminescence intensity of FAPbI_3_/BAAc TA and FAPbI_3_/BAAc RT films increased by 32.2% and 54.6%, respectively. This indicates that BAAc modification at room temperature more effectively inhibits the non‐radiative decay of the surface. In addition, TRPLanalysis provides a more profound understanding of charge recombination through non‐radiative channels associated with surface defects. The corresponding lifetimes (Figure S2, Supporting Information) were obtained by double‐exponential fitting of TRPL spectra. The PL lifetime (τ) of the FAPbI_3_/BAAc RT film is 243.4 ns, significantly longer than that of the FAPbI_3_/BAAc TA film (83.88 ns). The extended τ strongly suggests a much slower charge recombination rate, attributed to the reduction of surface defects by the BAAc passivation at room temperature.^[^ ^33^ ^]^ Figure S2 (Supporting Information) shows the PL mapping of FAPbI_3_/BAAc before and after annealing. The PL mapping of FAPbI_3_/BAAc TA exhibits a notably lower PL intensity throughout the region compared to the unannealed sample (Figure S3, Supporting Information). In addition, the PL intensity is unevenly distributed in the scanned regime.^[^ ^34^ ^]^ It is suspected that, even if the 2D perovskite layer formed after annealing, the 2D perovskite composition is randomly distributed over the surface of the 3D perovskite.

X‐ray photoelectron spectroscopy (XPS) was used to further confirm the effect of BAAc annealing on the defect properties of FAPbI_3_‐based perovskites (Figure S4a, Supporting Information). The primary peaks of Pb 4f7/2 and Pb 4f5/2 in the unannealed FAPbI_3_ films are located at binding energies of 138.3 and 143.2 eV, respectively. In the annealed FAPbI_3_ film, two additional peaks at 136.4 and 141.3 eV appear, attributed to metallic lead, possibly present due to the absence of iodine.^[^ ^35^ ^]^ Simultaneously, the disappearance of the Pb^0^ peak is likely due to the effective repair of uncoordinated Pb^2+^ defects by C═O in acetate. These results are consistent with previous reports suggesting that carboxyl groups can effectively passivate perovskite films through the formation of coordination effects.

To gain a comprehensive understanding of the evolution of the energy level in FAPbI_3_/BAAc post‐annealing films, a thorough analysis of the energy level in the perovskite films was conducted via ultraviolet photoconductive spectroscopy (UPS) (Figure S4b,c, Supporting Information), followed by an assessment of the change in the valence band maximum (VBM).^[^ ^36^ ^]^ The energy level diagram of the entire device is shown in Figure S4d (Supporting Information). The VBM values of FAPbI_3_/BAAc TA and FAPbI_3_/BAAc RT films are −5.74 and −5.40 eV, respectively. This result indicates that the annealing of BAAc leads to a downshift of the energy level in the perovskite, resulting in an increased energy barrier for transferring holes from perovskites to the hole transport layer.

In order to investigate the effect of annealing on photovoltaic performance, the champion current density–voltage (*J–V)* curves of FAPbI_3_/BAAc TA and FAPbI_3_/BAAc RT are presented in Figure 1g. The highest *PCE*, *Voc*, circuit current density (*Jsc*), and filling factor (*FF*) are 22.14%, 1.17 V, 24.55 mA cm^−2^, and 77.07% respectively. In comparison, for the optimal unannealed device, the *PCE* is 24.76%, the *Voc* is 1.19 V, the *Jsc* is 25.61 mA cm^−2^, and the *FF* is 81.22%, representing notable enhancement in photoelectric performance. The improvement in the *Voc* and *FF* can be attributed to the improved homogenous passivation effect and enhanced energy band alignment achieved with BAAc RT.

**Figure 2:**
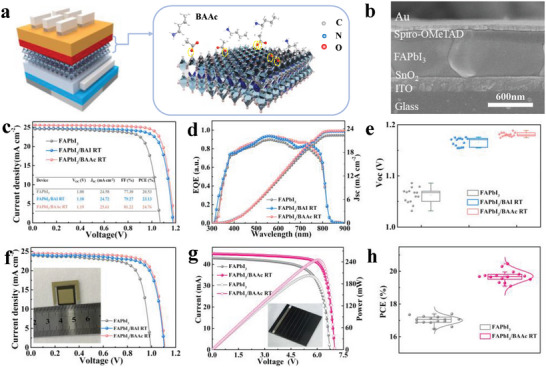
a) Device architecture (left part) and BAAc RT passivation diagram (right part). b) Cross‐section SEM images of the device. c) *J‐*‐*V* curves of the champion devices based on different perovskite thin films. d) EQE of the champion devices. e) The statistics of *Voc* for devices based on FAPbI_3_, FAPbI_3_/BAI, and FAPbI_3_/BAAc. f) *J‐*‐*V* curves of 1.32 cm^2^ champion device based on different perovskite films. g) *I‐*‐*V* curves of the with/without BAAc module with champion PCE, active area of 12 cm^2^. h) The statistics of PCE for devices based on different modules.

N‐butylammonium iodide (BAI) was introduced as a control to further investigate the effect of acetate in BAAc on FAPbI_3_ perovskite films. **Figure** **2**a shows the PSC structure of ITO/SnO_2_/FAPbI_3_/Spiro‐OMeTAD/Au film stacks, and Figure 2b displays the cross‐section of the fabricated device. The thickness of SnO_2_, FAPbI_3_, and Spiro‐OMeTAD layers is ≈ 35, 750, and 120 nm, respectively, consistent with previously reported high‐efficiency PSCs.^[^ ^37^ ^]^ As shown in Figure S5 (Supporting Information), the thickness of perovskite film did not change significantly before and after passivation. The *J–V* curve of the photovoltaic characteristics was tested with standard analog illumination at 100 mW cm^−2^ (AM 1.5G). To optimize the concentration of BAAc for device performance, Figure S6 (Supporting Information) presents the statistical distribution of *PCE*s at different concentrations, and Table S4 (Supporting Information) outlines the FAPbI_3_/BAAc RT performance parameters. The ideal concentration (volume fraction) for BAAc is determined to be 0.4%. For comparison, PSC devices without passivation and with a BAI passivation layer were fabricated. The BAI RT‐based device under reverse scanning (RS) exhibited a *PCE* of 23.13%, a *Voc* of 1.18 V, a *Jsc* of 24.72 mA cm^−2^, and an *FF* of 79.27%. In contrast, the optimal device with BAAc demonstrated a *PCE* of 24.76%, a *Voc* of 1.19 V, an *FF* of 81.22%, and a *Jsc* of 25.61 mA cm^−2^. The photovoltaic performance significantly improved after BAI RT and BAAc RT treatment compared to the original device (*PCE* of 20.53%, *Voc* of 1.08 V, *FF* of 77.30%, and *Jsc* of 24.58 mA cm^−2^). The integrated current densities, measured by external quantum efficiency (EQE) in Figure 2d, for the three perovskite devices, are 23.50, 23.31, and 22.34 mA cm^−2^, respectively. Notably, FAPbI_3_/BAAc RT devices exhibited low *J–V* curve hysteresis during forward and reverse scanning (Figure S7, Supporting Information). Table S5 (Supporting Information) summarizes the performance indicators of the champion device, and Figure S8a (Supporting Information) displays the statistical distribution of performance parameters for 15 units of each device. It confirms substantial improvement in the average *PCE* of PSCs after using the BAAc RT and BAI RT layers. In particular, the average *Voc* of the BAI RT‐ and BAAc RT‐based devices increased by ≈ 100 ± 10 and 130 ± 7 mV, respectively, compared to the control device (Figure 2e). BAAc RT‐treated perovskite devices exhibited a higher *Voc* (1.19 V) compared to other previously reported FAPbI_3_ PSCs (Figure S9 and Table S5, Supporting Information).

A large‐area device with an active area of 1.32 cm^2^ (1.1 cm × 1.2 cm) was further fabricated to explore the potential of scalable manufacturing strategies. As illustrated in Figure 4f, BAAc RT demonstrated a remarkable *PCE* of 21.22% in the large‐sized device. Table S6 (Supporting Information) provides a summary of the performance metrics for the champion device. To demonstrate the scalability of the deposition strategy for PSCs, Figure S10 (Supporting Information) shows a mini‐module (5 cm × 5 cm, active area 12 cm^2^, with scribing widths for P1, P2, and P3 lines at 43, 67, and 76 µm, respectively) consisting of six subcells. The current–voltage (*I–V)* characteristics in Figure 2g demonstrate the notable enhancement in the photovoltaic performance of the BAAc RT thin‐film‐based mini‐module, with a *PCE* of 20.47%, an *FF* of 77.6%, a *Voc* of 7.01 V, and an *Isc* of 45 mA (Table S7, Supporting Information). These values significantly surpass those of the control device (*PCE* = 17.39%, *FF* = 71.4%, *Voc* = 6.78 V, and *Isc* = 42.98 mA). Figure 2h summarizes the statistical analysis of PCEs from 15 mini‐modules, showing the excellent repeatability of the BAAc RT‐based mini‐modules. In summary, the performance of devices based on BAAc RT has demonstrated substantial improvement.

**Figure 3:**
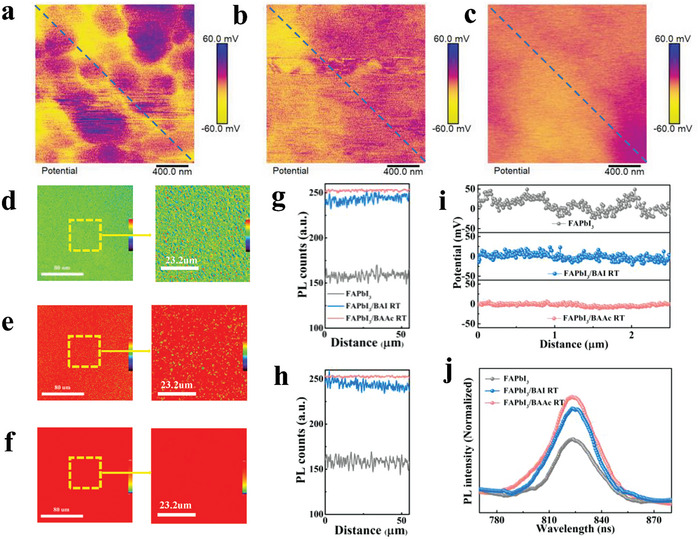
KPFM measurements of a) Control b) BAI RT and c) BAAc RT‐Passivated films. PL mapping of d) FAPbI_3_, e) FAPbI_3_/BAI RT, and f) FAPbI_3_/BAAc RT perovskite films (scale bar 80 µm (left) and 23.2 µm (right)). The right figure (scale bar 23.2 µm) corresponds to changes in PL intensity in the g) vertical and h) horizontal directions. i) Line profile of KPFM. j) Steady‐state PL curves of the perovskite films.

Exploring the impacts of BAI and BAAc on perovskite morphology is pivotal for gaining a comprehensive understanding of their passivation effects. The films comprised of FAPbI_3_, FAPbI_3_/BAI RT, and FAPbI_3_/BAAc RT were analyzed using atomic force microscopy (AFM) and PL mapping. Additionally, the surface morphology and surface potential uniformity in the BAI RT‐ or BAAc RT‐treated perovskite films were studied by Kelvin Probe Force Microscopy (KPFM). The topographical analysis, depicted in Figure S11 (Supporting Information), reveals that the root mean square roughness (RMS) of FAPbI_3_/BAI RT and FAPbI_3_/BAAc RT samples is 15.1 and 10.9 nm, respectively, indicating a reduction in surface RMS compared to the control perovskite film (18.5 nm). It is clear that the BAAc RT treatment results in a smoother perovskite surface. In **Figure** **3**a–c, the KPFM measurements illustrate a notable reduction in surface potential heterogeneity for films subjected to BAI RT and BAAc RT treatments. The BAAc RT passivated film exhibits uniform surface potential a potential change of 10 mV, whereas the FAPbI_3_ film show a surface potential change close to 80 mV. Notably, this regulation of the surface potential improves the *Voc* by mitigating non‐radiative recombination and enhancing the Quasi‐Fermi level splitting.^[^ ^38^ ^]^

Steady‐state PL spectra and PL mapping were used to investigate the influence of surface reconstruction on carrier transport dynamics. All samples were prepared on a silicon chip. PL mapping, as a reliable visualization technique for interfacial recombination, reveals in Figure 3d–f that the PL intensity of the BAAc RT passivation film consistently surpasses that of the control film (FAPbI_3_) and the BAI RT passivation film, indicating effective suppression of non‐radiative recombination.^[^ ^39^ ^]^ For a more intuitive representation of the change in PL intensities, horizontal and vertical intensity profiles were extracted from the PL mapping (Figure 3g,h). Compared to the control films, the BAAc RT films exhibited a stronger PL intensity with minimal intensity fluctuation, highlighting the efficacy of the ionic liquid as a passivation material. The PL results align with the PL mapping findings, showcasing a substantial increase in the PL intensity for the BAAc RT‐treated samples, almost doubling compared to the control film. The increased PL intensity signifies a lower trap density in the BAAc RT‐treated perovskite film.^[^ ^40^ ^]^ The collective evidence from PL mapping underscores the capability of BAAc RT treatment to deliver more uniform passivation of the perovskite surface, rendering it particularly suitable for large‐area perovskite thin films. Conductive Atomic Force Microscopy (c‐AFM) measures can further verify that the overall uniformity of perovskite films is caused by the comprehensive passivation of BAAc RT. The c‐AFM measurement results (Figure S12, Supporting Information) show that the photocurrent change of FAPbI_3_ single crystal is 3.07 pA, while the photocurrent change of BAAc‐based perovskite film in single crystal and at the grain boundary is <1.9 pA, indicating that the grain domain distribution or surface uniformity after treatment with the ionic liquid.

X‐ray diffraction (XRD) measurements were employed to analyze the crystal structures of the perovskite films deposited on ITO/SnO_2_ substrates. The XRD patterns of the three films in Figure S13 (Supporting Information) reveal distinct diffraction peaks at 14.0° and 28.3°, corresponding to the (100) and (200) perovskite crystal planes, respectively. Notably, the film treated with BAAc RT exhibits the highest intensity at the (100) peak compared to the other samples, indicating improved crystallinity of the perovskite films. More importantly, the complete elimination of the PbI_2_ signal at 12.6° by the BAAc RT passivation,^[^ ^41^ ^]^ affirming the reaction between the ionic liquid and PbI_2_.^[^ ^42^, ^43^ ^]^ XPS spectra were collected to gain insight into the by‐products in order to delve deeper into the passivation mechanism of BAAc. Figure S14b (Supporting Information) illustrates a strong C═O signal (288.1 eV), confirming the presence of BAAc on the surface of the perovskite. Furthermore, the Pb 4f peak shifts toward a lower binding energy upon interaction with BAAc and uncoordinated Pb^2+^. Uncoordinated Pb^2+^ is known to act as a non‐radiative recombination center, leading to a decrease in the Voc and FF. In addition, it also confirms that the introduction of BAAc can effectively mitigate the hysteresis effect in the device.^[^ ^35^, ^42^ ^]^ This interaction can inhibit the decomposition of perovskites, thereby restraining the formation of metallic Pb^0^, as evidenced by the reduced occurrence of Pb^0^ compared to the control (Figure S14c, Supporting Information).^[^ ^44^ ^]^

The energy levels of the perovskite films were assessed by UPS and ultraviolet–visible spectroscopy (UV–vis). As depicted in Figure S14a (Supporting Information), the absorption wavelength range of FAPbI_3_, FAPbI_3_/BAI RT, and FAPbI_3_/BAAc RT films is below 850 nm. The tau‐plot evaluation in Figure S15b (Supporting Information) indicates that the extracted bandgap of the FAPbI_3_ film is 1.53 eV, whereas that of the passivated FAPbI_3_ film is 1.52 eV. The calibrated UPS spectrum and energy level diagram (Figure S16, Supporting Information) reveal that the band structure of the passivated perovskite film exhibits better alignment with other functional layers.^[^ ^45^ ^]^ Therefore, the introduction of BAAc onto the perovskite film proves effective in passivating the uncoordinated Pb^2+^ defects, facilitating carrier transfer and resulting in a higher *Voc*.

Space‐charge‐limited current (SCLC) measurements were performed by fabricating hole‐only devices to elucidate the trap density (N_t_) and hole mobility, as depicted in Figure S17a (Supporting Information). The dark *I–V* curves, presented in Figure S17b,c (Supporting Information), reveal that the N_t_ values for FAPbI_3_/BAI and FAPbI_3_/BAAc RT perovskite devices decrease to 1.03 × 10^15^ and 0.93 × 10^15^ cm^−3^, respectively, in contrast to the trap density of the original device (1.68 × 10^15^ cm^−3^). The reduction in trapping density suggests the effectiveness of BAAc RT treatment in passivating surface defects.^[^ ^46^ ^]^ The hole mobility of the original perovskite and the films passivated with BAI RT and BAAc RT is determined to be 1.04 × 10^−3^, 2.49 × 10^−3^, and 3.07 × 10^−3^ cm^2^ V^−1^ s^−1^, respectively. The nearly threefold increase in hole mobility observed in the BAAc RT‐capped perovskite films confirms its favorable role in hole extraction.^[^ ^47^ ^]^

**Figure 4:**
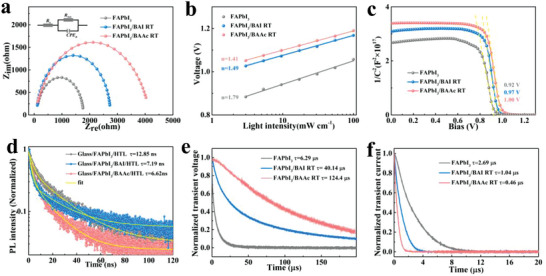
a) Nyquist plots of PSCs measured in the dark. b) *Voc* as a function of light intensity. c) Mott–Schottky plots of PSCs with different perovskite films. d) TRPL attenuation transient spectra of different active layer devices. e) Normalized transient photovoltage decay (TPV) and f) Normalized transient photocurrent decay (TPC) of PSCs.

To delve into the charge recombination dynamics at a device level, electrochemical impedance spectroscopy (EIS) measurements were conducted in the dark state to elucidate charge transport and recombination characteristics. The Nyquist diagrams for the three perovskite devices, shown in **Figure** **4**a, reveal a decrease in the series resistance (R_s_) of the device upon the introduction of BAAc RT. Furthermore, the introduction of BAI RT and BAAc RT increases the recombination resistance (R_rec_) in the low‐frequency region, with BAAc RT exhibiting a more pronounced effect in reducing interfacial recombination, which effectively retards the recombination of carriers within the device.^[^ ^48^ ^]^ Examining the variation of *Voc* with light intensities provides additional insights into the charge recombination mechanism in PSCs. Figure 4b shows that the slope of the control device, BAI RT‐, and BAAc RT‐based devices is 1.79, 1.49, and 1.41 kT q^−1^, respectively. The lower slope for devices processed with BAAc RT indicates fewer defect sites, leading to lower non‐radiative recombination losses, which is a crucial factor for achieving a higher *Voc*. Mott–Schottky measurements were carried out on perovskite devices (Figure 4c). The internal potential (V_bi_) of the control device is 0.92 V, which is lower than that of the devices treated by FAPbI_3_/BAI RT (0.97 V) and FAPbI_3_/BAAc RT (1.00 V). Notably, FAPbI_3_/BAAc RT exhibits the highest V_bi_ among the tested combinations of materials, enhancing charge separation and transport.^[^ ^49^ ^]^ This aligns with the synergistic effects of BAAc RT discussed earlier in minimizing defects and facilitating charge transfer dynamics. The calculated N_d_ values are 1.71 × 10^17^, 9.08 × 10^16^, and 7.51 × 10^16 ^cm^−3^ for the control, BAI RT‐, and BAAc RT‐treated devices, respectively. The reduction in charge accumulation observed in the BAAc‐treated device can be attributed to BAAc passivation, facilitating improved charge extraction.^[^ ^50^, ^51^ ^]^

TRPL measurements further confirm the accelerated charge transfer at the FAPbI_3_/BAAc RT/HTL interface.^[^ ^52^ ^]^ Compared with the FAPbI_3_/BAI RT/HTL film (average carrier lifetime = 7.19 ns) and the original perovskite film (average carrier lifetime = 12.85 ns) as shown in Figure 4d, the FAPbI_3_/BAAc RT/HTL film (average carrier lifetime = 6.62 ns) exhibits shortened carrier dynamics characterized by TRPL decay. To provide deeper insights into the enhancement conferred by BAAc, the charge transfer kinetics and charge recombination of different devices were assessed under open‐circuit conditions using transient photocurrent decay (TPV). The results (Figure 4e) demonstrate that, in comparison with pure FAPbI_3_ (6.29 µs) and FAPbI_3_/BAI RT (40.14 µs), FAPbI_3_/BAAc RT‐treated devices exhibit an extended charge recombination lifetime (τ_r_) (124.4 µs). This suggests that the electronic quality of perovskite films containing FAPbI_3_/BAAc RT is superior due to the lower defect concentration, consistent with the higher *Voc* of the corresponding device. Simultaneously, the impact of BAAc RT on charge transfer in the device was investigated through transient photocurrent decay (TPC) under short‐circuit conditions (Figure 4f). The resulting reduction in the charge transfer lifetime (τ_t_) to 0.46 µs for the BAAc RT‐based device suggests significant mitigation of interfacial defects and enhancement in the crystal orientation of FAPbI_3_/BAI RT films. The observed improvement in charge transfer can be attributed to the diminished presence of interfacial defects and the enhanced crystal orientation of FAPbI_3_/BAI RT films.^[^ ^53^ ^]^

Stability is a critical factor hindering the further development of PSCs. Changes in surface hydrophobicities were investigated through water contact angle characterization. The contact angles of FAPbI_3_/BAI RT (65.4°) and FAPbI_3_/BAAc RT films (71.1°) exhibit substantial increases compared to the control film (57.7°), indicating enhanced hydrophobicity in the perovskite films. This augmented hydrophobicity serves as an effective barrier against water vapor ingress into the perovskite. Images of the three perovskite films under different aging durations at 80% relative humidity (RH) are shown in Figure S18 (Supporting Information), directly reflecting the film alterations over time. Notably, the original FAPbI_3_ film degraded within 30 min, while the BAAc RT‐modified film remained nearly unchanged even after 12 h. The absorption strength of FAPbI_3_, FAPbI_3_/BAI RT, and FAPbI_3_/BAAc RT on glass under 80% ± 5% RH conditions are presented in Figure S19 (Supporting Information). The results reveal a significant reduction in the absorption intensity over time for FAPbI_3_ and FAPbI_3_/BAI RT, especially in the range of 400–600 nm, while the absorption intensity of the BAAc‐based perovskite film demonstrated greater stability.^[^ ^54^ ^]^ A comparative analysis of FAPbI_3_‐based n–i–p planar PSCs devices, as outlined in Table S3 (Supporting Information), underscores the superior stability and voltage characteristics of the BAAc RT‐based devices.

**Figure 5:**
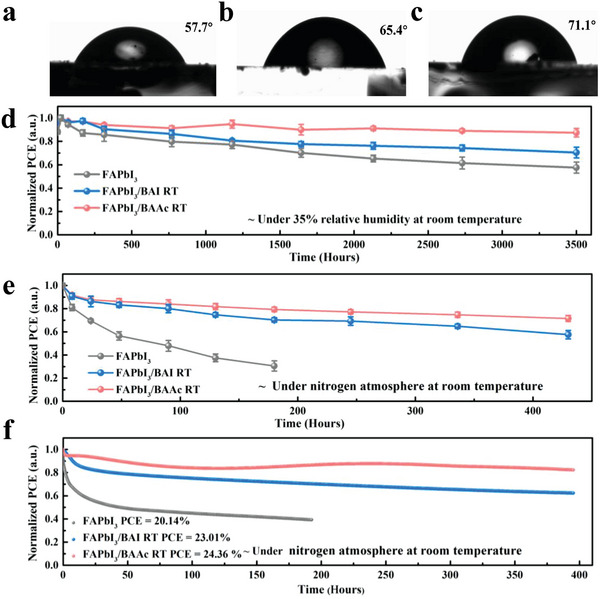
The water contact angle: a) FAPbI_3_, b) FAPbI_3_/BAI RT, and c) FAPbI_3_/BAAc RT films. d) Long‐term stability test (unencapsulated) at room temperature 35% relative humidity (RH). e) Long‐term stability test (unencapsulated) under continuous light in nitrogen environment (AM 1.5 G illumination). f) MPP tracking for PSCs without encapsulation under continuous AM 1.5 G illumination in nitrogen environment.

To further demonstrate the effect of BAAc RT on the device stability, the stability of the unpackaged device was monitored under ambient conditions (25 °C, 35% RH) for ≈ 3500 h, and the light stability of the unpackaged device was assessed during continuous light aging under N_2_ for ≈ 430 h (**Figure** **5**d,e). The initial and aged *J–V* curves of the device are shown in Figure S20 (Supporting Information). After humidity aging, BAI RT‐treated cells and BAAc RT‐treated cells retained 70.5% and 87.5% of their initial efficiency, respectively, whereas the control device only maintained 57.6%. In the continuous 400 h light aging experiment, the efficiency of the control device decreased to less than 40% of the initial value at 200 h, while the devices based on BAI RT and BAAc RT still maintained 60% and 80% of the initial efficiency after 400 h, respectively. In addition, the operational stability of devices using BAAc under MPP tracking was greatly improved, as presented in Figure 5f. After 400 h of continuous MPP tracking under lighting conditions, devices with BAAc RT and BAI RT retained 83.0% and 70.2% of their initial *PCEs*, respectively, while the control devices subjected to the same conditions for 200 h retained only 40%. All the above device results indicate that the BAAc RT surface treatment is a facile and effective approach to enhance the long‐term stability of PSCs.

## 3 Conclusion

In conclusion, an ionic liquid, BAAc, is introduced as a multifunctional interlayer material to improve the performance and stability of PSCs. The ultra‐thin BAAc modified layer is room‐temperature deposited on the perovskite layer (FAPbI_3_) to repair surface defects and optimize the energy level arrangement. The utilization of BAAc contributes to the enhancement of perovskite film morphologies, resulting in an exceptionally uniform surface. The BAAc RT leads to a noteworthy reduction in the number of uncoordinated surface defects in FAPbI_3_ perovskite films, thereby reducing defect densities, slowing down the charge‐trapping process, and inhibiting the non‐radiative complexation pathway. The *PCE* of the devices treated by BAAc RT achieves 24.76%, with a *Voc* value reaching as high as 1.19 V. Whether subjected to aging at 35% RH for 3500 h or MPP tracking for 400 h under continuous lighting, BAAc RT‐based PSCs consistently maintain 80% of the initial efficiency. Moreover, the *PCE* of perovskite solar mini‐modules based on BAAc RT thin films reaches 20.47%, underscoring the effectiveness of this method for the fabrication of large‐area, uniform, and high‐quality perovskite thin films.

## Conflict of Interest

The authors declare no conflict of interest.

## Supporting information

Supporting Information

## Data Availability

The data that support the findings of this study are available from the corresponding author upon reasonable request.
