# Supplementary material for: Room Temperature Ionic Liquid Capping Layer for High Efficiency FAPbI3 Perovskite Solar Cells with Long‐Term Stability
Source: Adv Sci (Weinh). 2024 Mar 13;11(19):2400117. doi: 10.1002/advs.202400117 (PMC11109663; doi:10.1002/advs.202400117)
Supplement: Supplementary file 1 — Supporting Information [file ADVS-11-2400117-s001.pdf]

ADVANCED SCIENCE
Open Access

Supporting Information

for *Adv. Sci.*, DOI 10.1002/advs.202400117

Room Temperature Ionic Liquid Capping Layer for High Efficiency $FAPbI_3$ Perovskite Solar Cells with Long-Term Stability

Qiang Lou, Xinxin Xu, Xueqing Lv, Zhengjie Xu, Tian Sun, Liwen Qiu, Tingting Dai, Erjun Zhou, Guijun Li, Tong Chen, Yen-Hung Lin and Hang Zhou**

Supplementary information for

Room temperature ionic liquid capping layer for high efficiency $FAPbI_3$ perovskite solar cells with long-term stability

Qiang Lou,[a] *Xinxin Xu,*[a] *Xueqing Lv,*[a] *Zhengjie Xu,*[a] *Tian Sun,*[a] *Liwen Qiu,*[a] *Tingting Dai,*[b] *Erjun Zhou,*[b]* *Guijun Li,*[c] *Tong Chen,*[a] *Yen-Hung Lin,*[d] *Hang Zhou*[a]*

a School of Electronic and Computer Engineering, Peking University Shenzhen Graduate School, Shenzhen 518055, China.

b CAS Key Laboratory of Nanosystem and Hierarchical Fabrication, CAS Center for Excellence in Nanoscience, National Center for Nanoscience and Technology, Beijing 100190, China;

c Key Laboratory of Optoelectronic Devices and Systems of Ministry of Education and Guangdong Province, College of Physics and Optoelectronic Engineering, Shenzhen University, Shenzhen 518060, China

d Department of Electronic and Computer Engineering, The Hong Kong University of Science and Technology Hong Kong SAR 999077, P. R. China

*Corresponding author: zhouh81@pkusz.edu.cn; zhouej@nanoctr.cn.

Experimental Section

Materials. Formamidinium Iodide (FAI, 99.9%), Lead Iodide (PbI_2, 99.99%), Methylammonium Chloride (MACl, 99.5%), 2,2",7,7"-Tetrakis [N,N-di(4-methoxyphenyl)amino]-9,9"-Spiro-bifluorene (Spiro-OMeTAD, 99.8%), 4-Tert-Butylpyridine (4-TBP, 96%), Bis (trifluoromethylsu-lfonyl)-Imide Lithium Salt (Li-TFSI, 99%). The above materials were purchased from Xi'an Polymer Optical Technology Co., Ltd. Dimethylsulfoxide (DMSO, 99%), N, N-dimethylformamide (DMF, 99%), Isopropanol (IPA, 99%) were purchased from Sigma-Aldrich. Tin oxide aqueous solution (SnO_2:15% mass in H_2O colloidal dispersion with a few organic solvents) was purchased from Alfa Aesar. Au (99.999%) was purchased from ZhongNuo Advanced Material (Beijing) Technology Co., Ltd.

Preparation of $FAPbI_3$ single crystal. 461 mg PbI_2 and 172 mg FAI were dissolved in 700 ul GBL and stirred at 60 °C for 6 hours. The fully dissolved solution was heated at 150 °C for 3 hours, and a large number of black broken crystals appeared. Use acetonitrile and ether to clean three times successively, and put the cleaned single crystals into a vacuum drying oven at 90 °C for 30 min.

Precursor preparation The ETL solution was prepared by diluting 15% of the nano-dispersed tin dioxide solution to 10% with deionized water. The perovskite precursor solution was obtained by mixing 886 mg $FAPbI_3$ single crystal and 17.6 mg MACl in 200 ul DMSO and 800 ul DMF overnight. The passivation layer precursor solution was obtained by dissolving IPA with 5 mg BAI or dispersing BAAc with different concentrations in IPA. 72.3 mg Spiro-OMeTAD was dissolved in 1 mL chlorobenzene, then 28.5 μL 4-TBP was added, stirring for 30 min, 17.5 μL Li-TFSI (520 mg Li-TFSI dissolved in 1 mL acetonitrile) was added. Stir at room temperature overnight to obtain HTL solution.

Thin Films and Devices Fabrication. Deionized water, acetone and isopropyl alcohol were used to clean the substrate for 20 minutes respectively, and then the ITO (sheet resistance ≈8 Ω, transmittance ≈94%) was cleaned by ultrasonic wave. The cleaned substrate is treated with UV-O for 30 min. After cooling to room temperature at 6500 r and annealing at 150 °C for 5 min, 10 wt% tin dioxide was spun onto the substrate. The SnO_2-coated base was then treated with UV-O for 1 hour, and the sample was quickly transferred to a nitrogen-atmosphere glove box. The perovskite precursor solution was spin coated on ITO/SnO_2 at a speed of 40 s 4000 r, and 1 ml Ethyl ether was quickly added to ITO/ SnO_2 at 10 s as an antisolvent. The spinning film was annealed at 150 °C for 10 min. To prepare HTL, the pre-prepared Spiro-OMeTAD precursor was spun onto calcium (5000 rpm, 15 s) on the titanite film, and gold electrodes of about 100 nm were deposited on the HTL by vacuum thermal evaporation. The preparation process of all PSCs was the same, and the effective area was 0.04 cm^2.

Characterization and measurements: The light source used in the light test involved in the article is a 450-watt xenon lamp (oriel solar simulator). The *J-V* curve of PSC is measured under a light source calibrated to 100 MW cm^{-2} with a silicon reference cell at room temperature. The EQE spectrum was recorded by the solar cell quantum efficiency test system (Enlitechnology, Taiwan, China). The Chi660e electrochemical measurement workstation was used to complete the measurement of space charge limited current (SCLC) and Mot-Schottky (M-S) and conductivity under dark conditions. The UV absorption spectrum of the film was measured in the wavelength range of 200-1000 nm by an ultraviolet-vis spectrophotometer (UV-2450, Japan). The UPS spectra were collected on the X-ray Photoelectron Spectrometer (Thermo Fisher, ESCALAB 250Xi, UK). The SEM image was obtained by a scanning electron microscope (ZEISS SUPRA 55, Germany) at

an accelerating voltage of 5 kV. Bruker Atomic Force Microscope (AFM) (MDTC-EQ-M16-01) is used for Kelvin Probe Force Microscope (KPFM) measurement. The film thickness measurements required for calculation are all measured with a profilometer (DEKTAK XT, Germany). The water contact angle of the film is measured using a contact angle measuring instrument (MDTC-EQ-M07-01, KRUSS DSA30). GIWAXS measurements were carried out with a Xeuss 2.0 SAXS/WAXS laboratory beamline using a Cu X-ray source (8.05 keV, 1.54 Å) and a Pilatus3R 300 K detector. The incidence angle is 0.3°. The steady-state PL spectra were recorded on Horiba jobin Yvon. The incident light at 510 nm forms glass surface as photon excitation. The time-resolved fluorescence was measured and analyzed by FLS 980 fluorescence spectrometer of Edinburgh instrument, and the time-resolved PL decay excitation was detected. XRD was conducted on the samples with the layer stack of ITO/$FAPbI_3$ using a Bruker D2Phaser system with Cu-Kα radiation (λ = 1.5405 Å) in Bragg–Brentano configuration using a LynxEye detector. TPV decay and TPC decay measurements were performed using a modular test bench (Arkeo, Cicci research s.r.l.) with white light bias generated by a diode array to simulate operating conditions of 0.5 solar bias light. A pulsed red dye laser pumped by a nitrogen laser was used as the perturbation source with a pulse width of 4 ns and a repetition frequency of 10 Hz. Open circuit voltage and short circuit current were measured on 1 MΩ and 50 Ω resistors and recorded on a digital oscilloscope (Tektronix DPO 4104B).

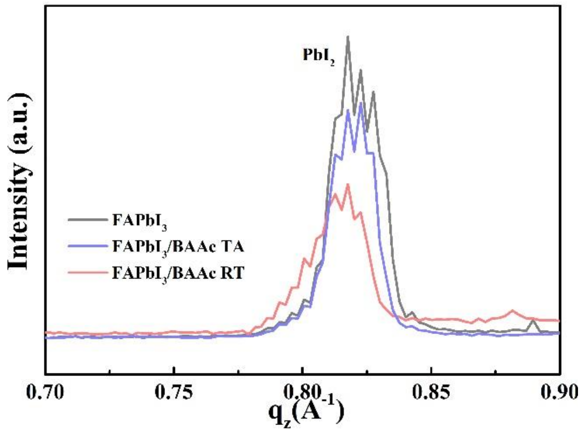

Figure S1 Cut-lines of GIWAXS intensities along the q_z axis.

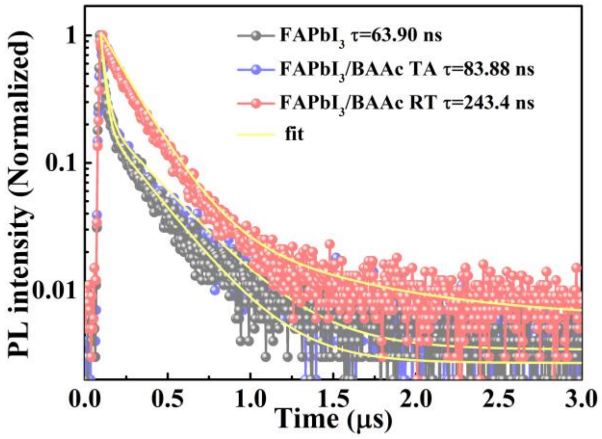

Figure S2 TRPL spectral curve.

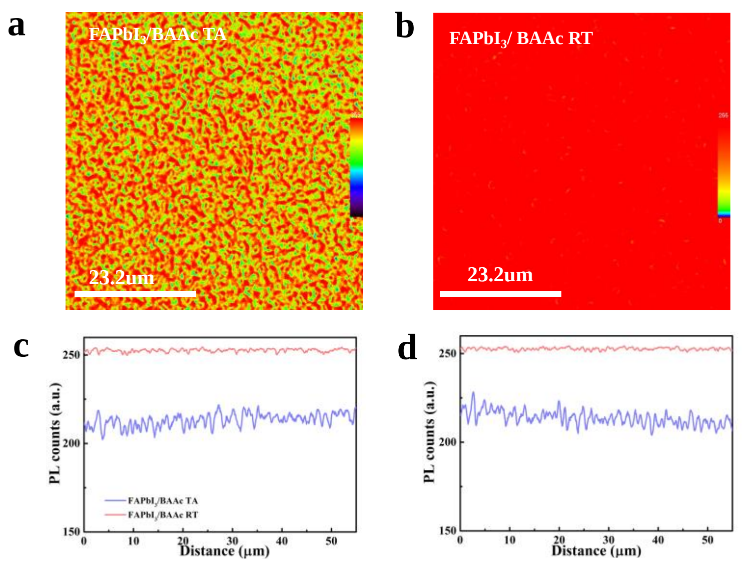

Figure S3 PL mapping of (a) $FAPbI_3$/BAAc TA and (b) $FAPbI_3$/BAAc RT perovskite films. The figure (scale bar 23.2μm) corresponds to changes in PL intensity in the (c) vertical and (d) horizontal directions.

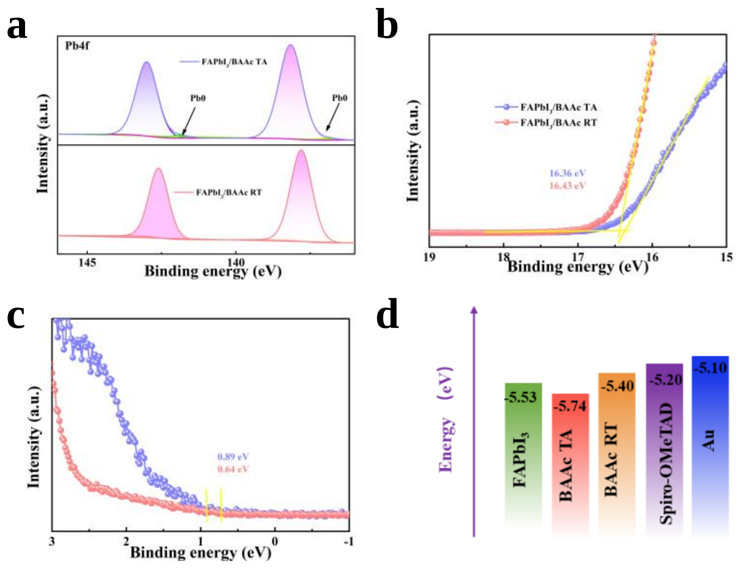

Figure S4 (a) XPS spectra of $FAPbI_3$/BAAc TA and $FAPbI_3$/BAAc RT films of Pb4f. Normalized ultraviolet photoelectron spectroscopy (UPS) showing (b) cutoff energy and (c) onset energy. (d) Energy level diagrams of PSCs.

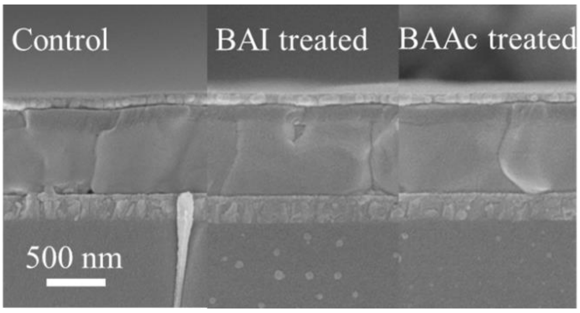

Figure S5 Cross-sectional SEM image of the PSCs (ITO/SnO_2/perovskite/ Spiro-OMeTAD/Au.

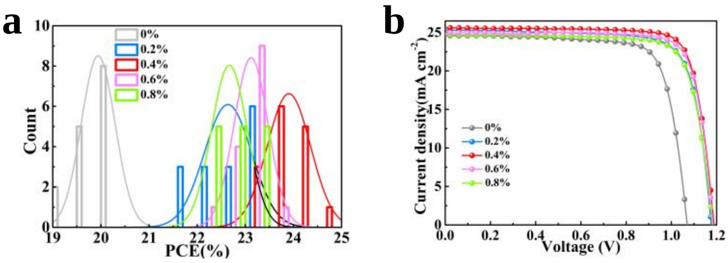

Figure S6 (a) PCE histograms of PSCs with different BAAc concentrations. (b) J-V curve of PSC based on different concentrations of BAAc.

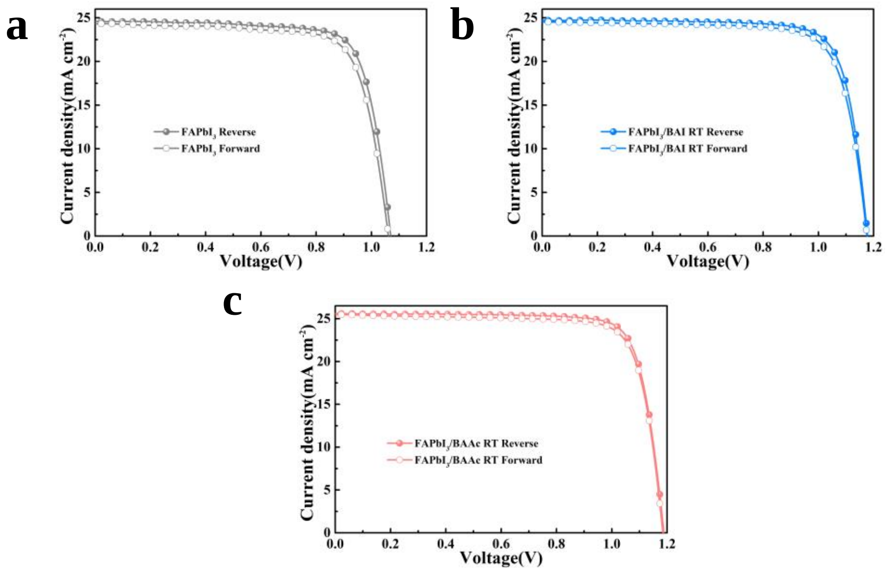

Figure S7 J-V curves of the champion devices based on different perovskite thin films.

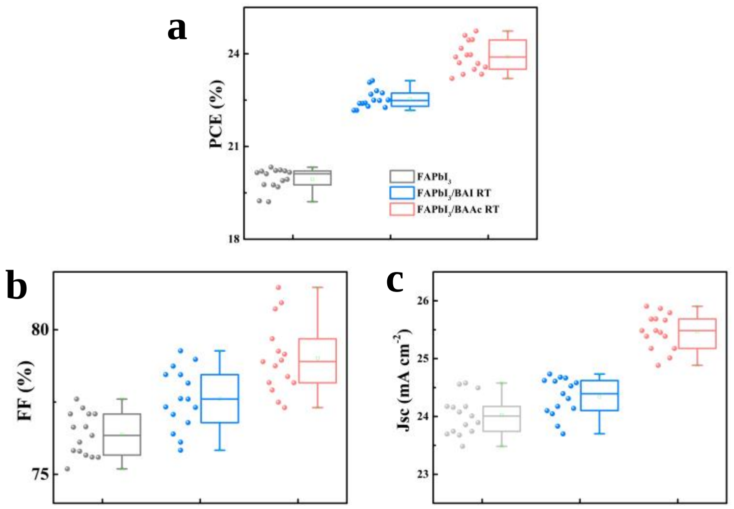

Figure S8 The statistics of (a) PCE, (b)FF, (c) Jsc for devices based on $FAPbI_3$, $FAPbI_3$/BAI and $FAPbI_3$/BAAc.

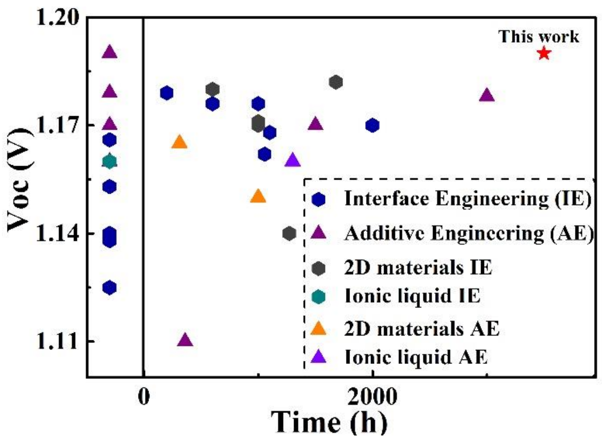

Figure S9 Statistical diagrams of voltage and humidity stability of n-i-p planar PSCs devices (PCE > 23%) based on $FAPbI_3$ (Detailed parameters are shown in Table S3)

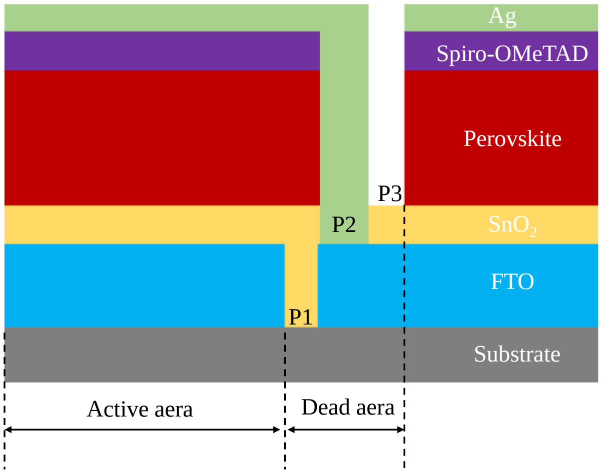

Figure S10 Schematic of a typical module.

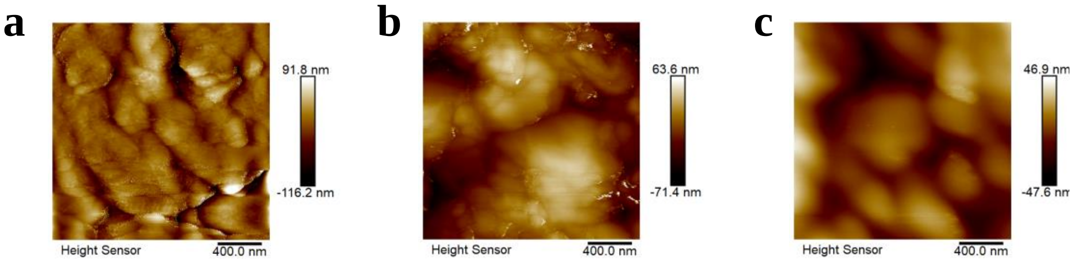

Figure S11 AFM of (a) $FAPbI_3$, (b) $FAPbI_3$/BAI RT and (c) $FAPbI_3$/BAAc RT films

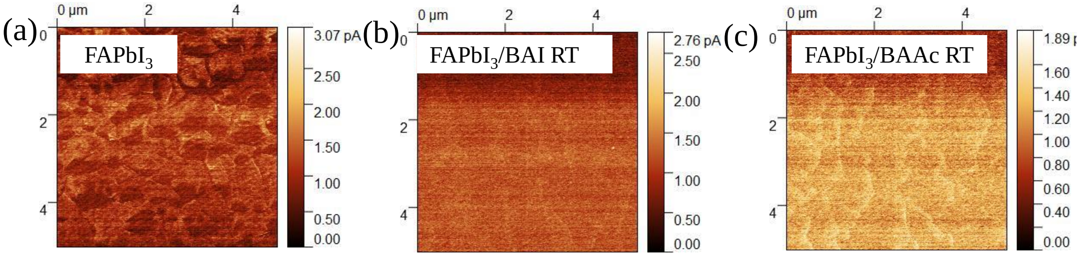

Figure S12 c-AFM of (a) ITO/SnO_2/$FAPbI_3$, (b) ITO/SnO_2/$FAPbI_3$/BAI RT and (c) ITO/SnO_2/$FAPbI_3$/BAAc RT.

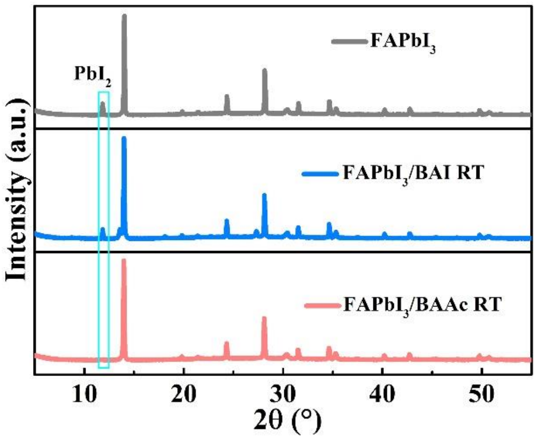

Figure S13 XRD-pattern of different perovskite thin films.

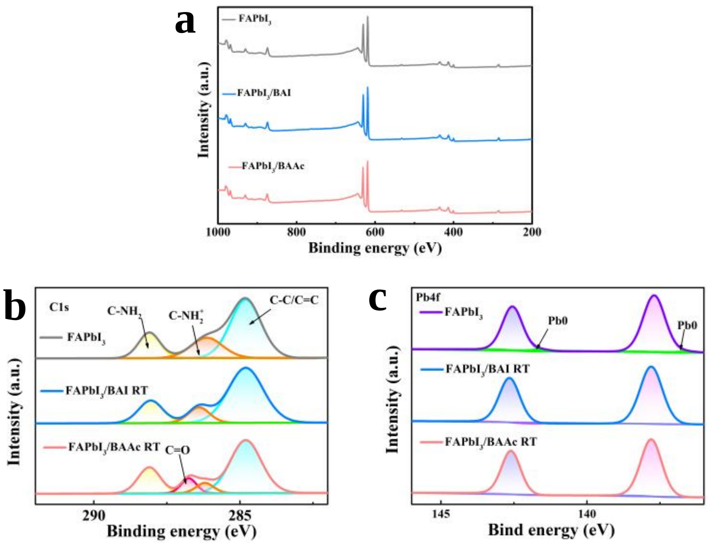

Figure S14 (a) XPS spectra of $FAPbI_3$, $FAPbI_3$/BAI RT and $FAPbI_3$/BAAc RT films. XPS spectra of $FAPbI_3$, $FAPbI_3$/BAI RT and $FAPbI_3$/BAAc RT films of (b) C1s and (c) Pb4f.

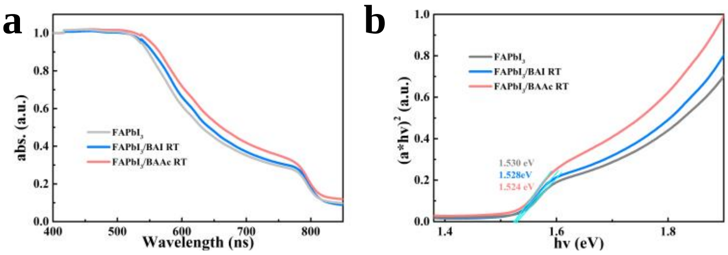

Figure S15 (a) The absorption spectra for different perovskite films. (b) Tauc plots of different perovskite thin films.

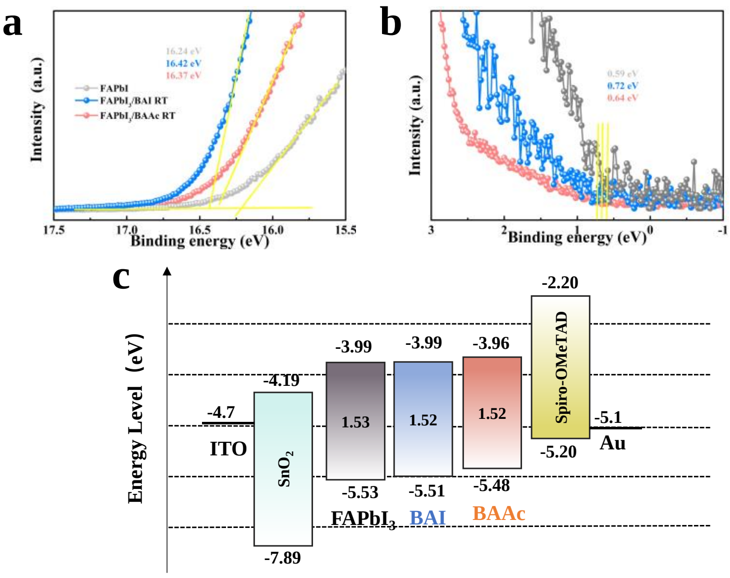

Figure S16 Normalized ultraviolet photoelectron spectroscopy (UPS) showing (a) onset energy and (b) cutoff energy. (c) Energy level diagrams of PSCs.

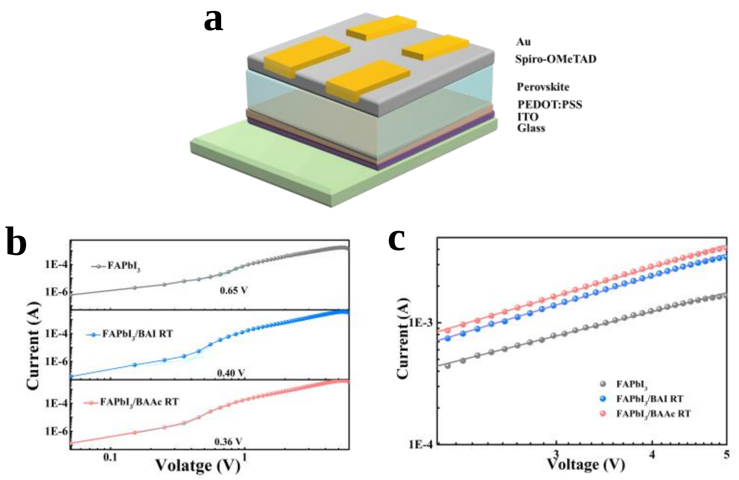

Figure S17 (a) ITO/PEDOT:PSS/perovskite/Spiro-OMeTAD/Au pure hole devices. (b) and (c) Dark-state I–V curves were obtained for a pure hole device of different perovskite.

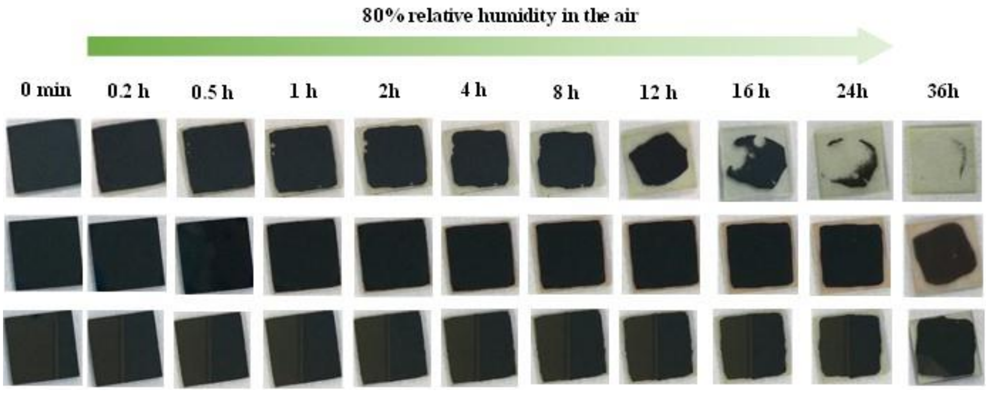

Figure S18 Photographs of control, BAI, and BAAc modified perovskite films aged in ambient air with a relative humidity of ≈80%.

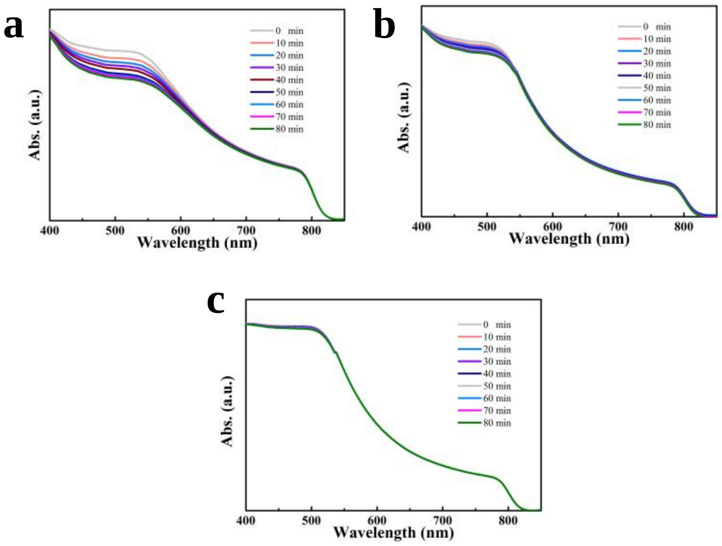

Figure S19 The absorption spectra for (a) $FAPbI_3$, (b) $FAPbI_3$/BAI and (c) $FAPbI_3$/BAAc film in 80% ± 5% RH atmosphere for 80 min.

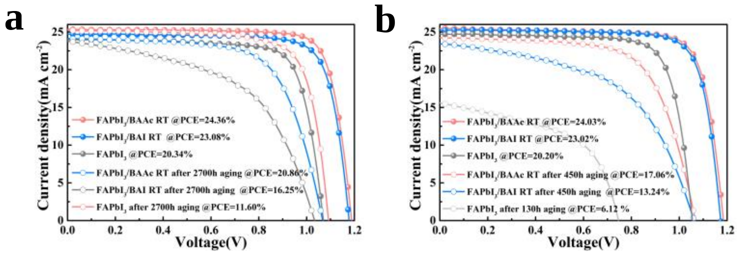

Figure S20 (a) Initial and aged *J-V* curves (long-term stability test at room temperature 35% relative humidity). (b) Initial and aged *J-V* curves (long-term stability test under continuous light in nitrogen environment).

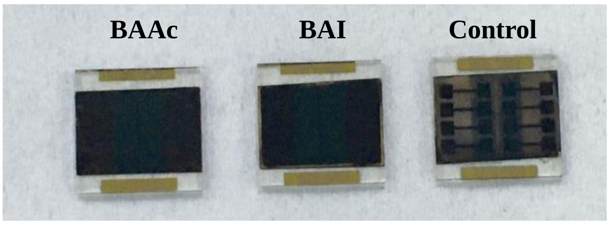

Figure S21 Photographs of control, BAI and BAAc modified devices were subjected to continuous light aging for 400 hours in nitrogen ambient air.

Table S1 Parameters used fit biexponential function to TPRL curves measured for pristine and modified HTL films. The average recombination lifetime (τ_{ave}) was estimated from the fitted data by biexponential decay function, the equation is as follows:

$$\tau_{ave} = \frac{\sum A_i \tau_i^2}{\sum A_i \tau_i}$$

where τ_i is decay times and A_i is amplitudes.

Devices	τ_1 (ns)	A_1	τ_2 (ns)	A_2	τ (ns)
$FAPbI_3$	22.95	0.91	260	0.19	63.90
$FAPbI_3$/BAAc TA	28.40	0.72	305.8	0.18	83.88
$FAPbI_3$/BAAc RT	104.5	0.81	778.7	0.21	243.4

Table S2 Performance summary of the PSCs with/without annealing process.

Devices	V_{OC} (V)	J_{SC} (mA cm^{-2})	FF (%)	PCE (%)
$FAPbI_3$/BAAc TA	1.17	24.55	77.07	22.14
$FAPbI_3$/BAAc RT	1.19	25.61	81.22	24.76

Table S3 Summary of character and humidity stability of $FAPbI_3$-based n-i-p planar PSCs devices.

Device structure	V_{OC} (V)	PCE (%)	Humidity stability	Year	Ref.
**ITO/SnO_2/Li_2CO_3/$FAPbI_3$/Spiro-OMeTAD/Au**	1.140	23.5	None	2022	[1]
**ITO/SnO_2/FSA/$FAPbI_3$/Spiro-OMeTAD/Au**	1.166	24.1	None	2022	[2]
**ITO/TiO_2/SnO_2/$FAPbI_3$/$CsPbBr_3$ NC /Spiro-OMeTAD/Au**	1.153	23.66	None	2022	[3]
**FTO/c-TiO_2/mp-TiO_2/$FAPbI_3$/ FPEAI/Spiro-OMeTAD/Au**	1.182	23.18	In ambient air with a relative humidity of 25-40% at RT in dark condition, the outperforming 3D/2D devices remain 88% of their initial performance after 1680 h aging test time.	2022	[4]
**ITO/SnO_2/$FAPbI_3$/P1/Spiro-OMeTAD/Au**	1.168	23.51	Stored under 30 ± 5% relative humidity (RH) at room temperature (25 °C), the PSCs show stability by retaining 92% of the initial efficiency after 1100 h aging.	2022	[5]
**ITO/SnO_2/$FAPbI_3$-RNH_3Cl/ Spiro-OMeTAD/Au**	1.190	25.08	None	2022	[6]
**ITO/SnO_2/$FAPbI_3$-TOP/Spiro-OMeTAD/Au**	1.170	24.90	None	2022	[7]
**ITO/SnO_2/$FAPbI_3$-MAAc/Spiro-OMeTAD/Au**	1.160	23.91	None	2022	[8]
**FTO/TiO_2/$FAPbI_3$/DIAI/Spiro-OMeTAD/Au**	1.176	24.13	The unencapsulated stability of devices was tested at a temperature of 25 °C and humidity of 45% in the air environment for 1000 h. The device maintained 94% of its initial efficiency.	2022	[9]
**FTO/c-TiO_2/mp-TiO_2/$FAPbI_3$-TTDA /Spiro-OMeTAD/Au**	1.11	23.35	Unencapsulated solar cells were stored in a humidity-controlled instrument for 360 h in the dark, with RH maintained at ≈ 60%, the cell retains≈96.5% of the initial PCE.	2022	[10]
**ITO/SnO_2/$FAPbI_3$-Cl-PACl/Spiro-OMeTAD/Ag**	1.14	23.03	Evaluated the environmental stability (R_h = 30 ± 5%, T = 25 °C) of the devices by tracking the efficiency for 1272 h, the PCE retains 88% of the initial PCE after 1272 h.	2022	[11]
**ITO/SnO_2/CL-PCBM/$FAPbI_3$/Spiro-OMeTAD/Au**	1.125	24.19	None	2022	[12]

**ITO/SnO_2/$FA_xMA_{1-x}PbI_3$/OACl/ Spiro-OMeTAD/Au**	1.171	24.97	The unencapsulated cells maintain 81% of their initial efficiencies after 1,000 h MPPT at about 55°C in a nitrogen-filled glove box	2022	[13]
**ITO/SnO_2/$FAPbBr_3$-$FAPbI_3$ /Spiro-OMeTAD/Au**	1.178	23.84	The unencapsulated PSC retains 91% of the initial PCE after 3000 h of storage at ≈30-50% relative humidity.	2023	[14]
**ITO/TiO_2/SnO_2/$FAPbI_3$-PAd/ Spiro-OMeTAD/Au**	1.150	25.02	The unencapsulated PSC retains over 90% of its initial efficiency after stored in RH:30 ± 5% for 1000 h.	2023	[15]
**ITO/SnO_2/$FAPbI_3$/DFBA/Spiro-OMeTAD/Au**	1.179	24.02	The unencapsulated PSC retains 70% of initial PCE after 200 h exposed to ambient environment (25 °C and 50 ± 5% relative humidity)	2023	[16]
**FTO/SnO_2/$FAPbI_3$/IPIE/Spiro-OMeTAD/Au**	1.160	24.12	None	2023	[17]
**FTO/SnO_2/$FAPbI_3$/H_2PC/Spiro-OMeTAD/Au**	1.138	24.03	None	2023	[18]
**ITO/SnO_2/$FAPbI_3$/PF/Spiro-OMeTAD/Au**	1.170	24.61	The encapsulated devices remained PCE of 90.7 % over 2000h, 90.2 % over 1200h of 65℃, 85 RH% and 65℃, 50 RH%, 1-sun illumination.	2023	[19]
**ITO/SnO_2/$FAPbI_3$/PAH/Spiro-OMeTAD/Au**	1.180	24.60	The PCE of unencapsulated device retains 89.2% of its initial efficiency after 600 h storage at room temperature and 40–50% humidity.	2023	[20]
**ITO/ SnO_2/$FAPbI_3$-RACl/Spiro-OMeTAD/Au**	1.179	25.73	None	2023	[21]
**FTO/ SnO_2/$FAPbI_3$-PYBA/Spiro-OMeTAD/Au**	1.170	24.76	Retained over 80% of its initial PCE after 1500 h under MPP tracking conditions. (relative humidity (RH) of 65 ± 5%, and temperature of 65 ± 2 °C)	2023	[22]
**FTO/TiO_2/$FAPbI_3$-PPTFB/Spiro-OMeTAD/Au**	1.190	24.00	None	2023	[23]
**ITO/SnO_2/FAFa/$FAPbI_3$/Spiro-OMeTAD/Au**	1.160	23.11	FAFa treated device can retain more than 97% of the initial efficiency in glove box for 1000 h and still can reserve 88% even after a further 300 h storage in the open air.	2023	[24]
**FTO/SnO_2/$FAPbI_3$/MACl/Spiro-OMeTAD/Au**	1.162	23.20	The device can retain more than 60% of the initial efficiency at 25 °C and under a relative humidity (RH) of 45−65% before and after measurements) for 1056 h.	2023	[25]

**FTO/SnO_2/$FAPbI_3$-PEA_2PbI_4/Spiro-OMeTAD/Au**	1.165	23.69	Tested the stability of devices in ambient air conditions (25 °C and 30 ± 10% relative humidity, in the dark). The unencapsulated device retained 93% of its initial efficiency after 312 h.	2023	[26]
**FTO/TiO_2/$FAPbI_3$/Rb-PAA/Spiro-OMeTAD/Au**	1.176	24.69	The PSCs are aged at 65°C under an ambient condition with an RH (relative humidity) of 30%. The PSCs with Rb-PAA passivation decreased by <10% after 600 h.	2023	[27]
**FTO/SnO_2/$FAPbI_3$/BABr/Spiro-OMeTAD/Au**	1.17	24.95	The device maintaining 96% of the initial PCE under 1000 hours of continuous white light at 100 mW cm^{-2} intensity in an atmosphere of ≈55°C N_2.		[28]
**ITO/SnO_2/$FAPbI_3$/BAAc/Spiro-OMeTAD/Au**	1.19	24.76	The unencapsulated device retained 87.5% of its initial efficiency after 3500 h at an external environment (25°C, 35% RH).	2023	This work

Table S4 Performance summary of the PSCs with different concentrations of BAAc aqueous solution.

v/v	V_{OC} (V)	J_{SC} (mA cm^{-2})	FF (%)	PCE (%)
0%	1.08	24.58	77.30	20.53
0.2%	1.18	25.29	77.74	23.20
0.4%	1.19	25.61	81.22	24.76
0.6%	1.19	24.97	80.96	24.06
0.8%	1.19	24.77	78.73	23.21

Table S5 Summary of the photovoltaic performances for the reverse and forward scans of the devices with or without passivation interlayers

Devices	V_{OC} (V)	J_{SC} (mA cm^{-2})	FF (%)	PCE (%)	HI (%)
$FAPbI_3$ RS	1.08	24.58	77.30	20.53	4.48
$FAPbI_3$ FS	1.07	24.32	75.44	19.64	
$FAPbI_3$/BAI RT RS	1.18	24.72	79.27	23.13	3.36
$FAPbI_3$/BAI RT FS	1.18	24.52	77.34	22.38	
$FAPbI_3$/BAAc RT RS	1.19	25.61	81.22	24.76	2.67
$FAPbI_3$/BAAc RT FS	1.19	25.43	79.69	24.12	

Table S6 Performance summary of the 1.32 cm^2 champion devices.

Devices	V_{OC} (V)	J_{SC} (mA cm^{-2})	FF (%)	PCE (%)
$FAPbI_3$	1.00	24.00	74.20	17.81
$FAPbI_3$/BAI RT	1.11	24.20	76.08	20.44
$FAPbI_3$/BAAc RT	1.12	24.54	77.20	21.22

Table S7 Performance summary of the mini-module with/without BAAc.

Devices	V_{OC} (V)	I_{SC} (mA)	FF (%)	PCE (%)
$FAPbI_3$	6.78	42.98	71.40	17.39
$FAPbI_3$/BAAc	7.01	45.00	77.66	20.47

Table S8 Charge density, Hole defects density and hole mobilities of different devices.

Devices	Charge density (cm^{-3})	Hole mobility (cm^2 V^{-1} s^{-1})	Hole defect density (cm^{-3})
$FAPbI_3$	1.71×10^{17}	1.04×10^{-3}	1.68×10^{15}
$FAPbI_3$/BAI RT	9.08×10^{16}	2.49×10^{-3}	1.03×10^{15}
$FAPbI_3$/BAAc RT	7.51×10^{16}	3.07×10^{-3}	0.93×10^{15}

Table S9 The parameters of glass/perovskite/HTL TPRL curve were fitted by fitting double exponential function.

Devices	τ_1 (ns)	A_1	τ_2 (ns)	A_2	τ (ns)
$FAPbI_3$	24.13	0.42	3.39	0.50	12.85
$FAPbI_3$/BAI RT	23.89	0.23	1.92	0.73	7.19
$FAPbI_3$/BAAc RT	16.73	0.30	1.65	0.61	6.62

Table S10 The fitting parameters for measured EIS results with different devices.

Devices	R_s (Ω)	R_{rec} (Ω)
$FAPbI_3$	103	1669
$FAPbI_3$/BAI RT	51	2788
$FAPbI_3$/BAAc RT	34	4130

Table S11 Performance summary of the fresh and aged devices.

Devices	V_{OC} (V)	J_{SC} (mA cm^{-2})	FF (%)	PCE (%)
$FAPbI_3$ Fresh	1.07	24.10	78.88	20.34
$FAPbI_3$ Aging	1.02	23.78	47.83	11.60
$FAPbI_3$/BAI RT Fresh	1.18	24.73	79.10	23.08
$FAPbI_3$/BAI RT Aging	1.06	24.01	63.85	16.25
$FAPbI_3$/BAAc RT Fresh	1.18	25.38	81.34	24.36
$FAPbI_3$/BAAc RT Aging	1.08	25.20	76.65	20.86

Table S12 Performance summary of the fresh and aged devices.

Devices	V_{OC} (V)	J_{SC} (mA cm^{-2})	FF (%)	PCE (%)
$FAPbI_3$ Fresh	1.05	24.71	77.86	20.20
$FAPbI_3$ Aging	0.75	15.47	52.75	6.12
$FAPbI_3$/BAI RT Fresh	1.17	25.25	77.93	23.02
$FAPbI_3$/BAI RT Aging	1.05	23.34	54.03	13.24
$FAPbI_3$/BAAc RT Fresh	1.19	25.50	79.19	24.03
$FAPbI_3$/BAAc RT Aging	1.06	24.25	66.37	17.06

Reference

[1] Y. Zhang, T. Kong, H. Xie, J. Song, Y. Li, Y. Ai, Y. Han, D. Bi, ACS Energy Letters, 7 (2022) 929-938.

[2] Z. Qin, Y. Chen, X. Wang, N. Wei, X. Liu, H. Chen, Y. Miao, Y. Zhao, Advanced Materials, 34 (2022).

[3] Y. Chen, N. Wei, Y. Miao, H. Chen, M. Ren, X. Liu, Y. Zhao, Advanced Energy Materials, 12 (2022).

[4] S. Hassan Kareem, M. Harjan Elewi, A. Muhson Naji, D.S. Ahmed, M. K. A. Mohammed, Chemical Engineering Journal, 443 (2022) 136469.

[5] Y. Meng, J. Zhang, C. Liu, K. Zheng, L. Xie, S. Bu, B. Han, R. Cao, X. Yin, C. Liu, Z. Ge, Advanced Functional Materials, 33 (2022).

[6] H.-S. Yun, H.W. Kwon, M.J. Paik, S. Hong, J. Kim, E. Noh, J. Park, Y. Lee, S. Il Seok, Nature Energy, 7 (2022) 828-834.

[7] H. Min, S.-G. Ji, S.I. Seok, Joule, 6 (2022) 2175-2185.

[8] L. Chao, Y. Xia, X. Duan, Y. Wang, C. Ran, T. Niu, L. Gu, D. Li, J. Hu, X. Gao, J. Zhang, Y. Chen, Joule, 6 (2022) 2203-2217.

[9] X. Du, J. Zhang, H. Su, X. Guo, Y. Hu, D. Liu, N. Yuan, J. Ding, L. Gao, S. Liu, Advanced Materials, 34 (2022) 2204098.

[10] R. Nie, W. Chu, Z. Li, H. Li, S. Chen, Y. Chen, Z. Zhang, X. Liu, W. Guo, S.I. Seok, Advanced Energy Materials, 12 (2022).

[11] Y. Huang, J. Liang, Z. Zhang, Y. Zheng, X. Wu, C. Tian, Z. Zhou, J. Wang, Y. Yang, A. Sun, Y. Liu, C. Tang, Z. Chen, C.C. Chen, Small Methods, 6 (2022).

[12] C. Ding, L. Yin, J. Wang, V. Larini, L. Zhang, R. Huang, M. Nyman, L. Zhao, C. Zhao, W. Li, Q. Luo, Y. Shen, R. Österbacka, G. Grancini, C.Q. Ma, Advanced Materials, 35 (2022).

[13] Y. Ge, H. Wang, C. Wang, C. Wang, H. Guan, W. Shao, T. Wang, W. Ke, C. Tao, G. Fang, Advanced Materials, 35 (2023) 2210186.

[14] J. He, D. Li, H. Liu, J. Xiang, J. Bai, Y. Ren, Z. Wang, M. Xia, X. Yin, L. Yuan, F. Zhang, S. Wang, Advanced Energy Materials, 13 (2023).

[15] P. Shi, Y. Ding, B. Ding, Q. Xing, T. Kodalle, C.M. Sutter-Fella, I. Yavuz, C. Yao, W. Fan, J. Xu, Y. Tian, D. Gu, K. Zhao, S. Tan, X. Zhang, L. Yao, P.J. Dyson, J.L. Slack, D. Yang, J. Xue, M.K. Nazeeruddin, Y. Yang, R. Wang, Nature, 620 (2023) 323-327.

[16] Y. Liu, J. Guo, Y. Long, H. Zhou, H.-Q. Wang, J. Song, Chemical Engineering Journal, 473 (2023)

145288.

[17] G. Qu, Y. Qiao, J. Zeng, S. Cai, Q. Chen, D. Wang, D. Khan, L. Huang, B. Xu, J. Chen, T. El-Assaad, Y.-G. Wang, D.V. McGrath, Z.-X. Xu, Nano Energy, 118 (2023).

[18] Y. Wang, Z. Shi, Y. Wang, Q.U. Khan, X. Li, L. Deng, Y. Pan, X. Zhang, Y. Yang, X. Yue, T. Hu, F. Liu, H. Wang, C. Li, K. Liu, W. Yuan, C. Cong, A. Yu, Y. Zhan, Advanced Materials, (2023) doi: 10.1002/adma.202302298.

[19] R. Xu, F. Pan, J. Chen, J. Li, Y. Yang, Y. Sun, X. Zhu, P. Li, X. Cao, J. Xi, J. Xu, F. Yuan, J. Dai, C. Zuo, L. Ding, H. Dong, A.K.Y. Jen, Z. Wu, Advanced Materials, (2023) 202308039.

[20] L. Liu, J. Tang, S. Li, Z. Yu, J. Du, L. Bai, X. Li, M. Yuan, T. Jiu, Advanced Functional Materials, 33 (2023) 202303038.

[21] J. Park, J. Kim, H.-S. Yun, M.J. Paik, E. Noh, H.J. Mun, M.G. Kim, T.J. Shin, S.I. Seok, Nature, 616 (2023) 724-730.

[22] R. Wang, X. Li, J. Qi, C. Su, J. Yang, S. Yang, M. Yuan, T. He, Advanced Materials, 35 (2023).

[23] Y. Du, Q. Tian, S. Wang, L. Yin, C. Ma, Z. Wang, L. Lang, Y. Yang, K. Zhao, S. Liu, Advanced Materials, (2023).

[24] Y. Wang, B. Zhou, M. Han, J. Zhao, R. Wang, J. Zhang, H. Ren, G. Hou, Y. Ding, Y. Zhao, X. Zhang, Nano Energy, 118 (2023).

[25] D.-H. Kang, S.-U. Lee, N.-G. Park, ACS Energy Letters, 8 (2023) 2122-2129.

[26] R. Zhi, C.-Q. Yang, M.U. Rothmann, H.-Q. Du, Y. Jiang, Y.-Y. Xu, Z.-W. Yin, Y.-P. Mo, W. Dong, G. Liang, U. Bach, Y.-B. Cheng, W. Li, ACS Energy Letters, 8 (2023) 2620-2629.

[27] C. Zhao, H. Zhang, M. Almalki, J. Xu, A. Krishna, F.T. Eickemeyer, J. Gao, Y.M. Wu, S.M. Zakeeruddin, J. Chu, J. Yao, M. Grätzel, Advanced Materials, 35 (2023).

[28] H. Wang, F. Ye, J. Liang, Y. Liu, X. Hu, S. Zhou, C. Chen, W. Ke, C. Tao, G. Fang, Joule, 6 (2022) 2869-2884.
